# Potential carbon emissions reduction from fleet schedule reconfiguration of China’s and India’s external routes

**DOI:** 10.1016/j.patter.2022.100614

**Published:** 2022-10-28

**Authors:** Qiang Cui, Yi-lin Lei, Ye Li

**Affiliations:** 1 School of Economics and Management, Southeast University, Nanjing, China; 2 School of Business Administration, Nanjing University of Finance and Economics, Nanjing, China

**Keywords:** fleet reconfiguration, emission reduction, aircraft costs, China-foreign routes, India-foreign routes

## Abstract

This article calculates the carbon emissions from China-foreign and India-foreign routes in 2019. The error rate of China’s and India’s external routes is about 2.75% and 5.21%, respectively. First, it obtains the aircraft with the least emission intensity in each distance segment. Then, we use the aircraft with the lowest emission intensity to replace the original aircraft and compare the emission reduction. The results show that after the aircraft is reconfigured, the CO_2_ of China-foreign and India-foreign routes has declined by 57.08% and 35.45%, respectively. Interestingly, the average aircraft costs of China-foreign and India-foreign routes also dropped 8.04% and 39.39%, respectively, after the reconfiguration. Therefore, fleet schedule reconfiguration is one of the most feasible ways for airlines to reduce emissions. However, for airlines with increased average aircraft costs, only when the carbon price is eight times the current level can they offset the increase in the average aircraft costs.

## Highlights

• Calculate the CO_2_ emissions of China’s and India’s external routes
• Discuss the potential carbon emissions reduction through fleet schedule reconfiguration
• Carbon emissions and aircraft costs have both declined
• Fleet schedule reconfiguration is feasible for airlines to reduce emissions and costs

## The bigger picture

The rapid development of the aviation industry has brought about a significant increase in emissions, and fleet optimization is one of the potential ways for airlines to reduce emissions. Based on the modified fuel percentage method (MFPM), this paper calculates the carbon emissions of China’s and India's external routes. It obtains the carbon emission intensity of each aircraft type at different distances, then uses the aircraft with the lowest emission intensity to replace the original aircraft and compare the emission reduction. This paper systematically discusses the emission reduction path of fleet reconfiguration and finds that it is feasible for airlines to reduce carbon emissions and costs.

## Teaser

For airlines, fleet optimization is one of the potential ways to reduce emissions. The results of China’s and India's external routes demonstrate that fleet reconfiguration is feasible for airlines to reduce carbon emissions and costs.

## Introduction

China and India are both populous countries, with a population of 1.411 billion in China and 1.38 billion in India in 2020.^1^ At the same time, both countries have one of the fastest-growing economies and aviation industries globally. According to the World Bank, in the decade before the coronavirus disease 2019 (COVID-19) (2010–2019), China's GDP growth averaged 7.68%, while India's was 6.66%. China's average air passenger traffic growth rate was 11.18%, while India's was 12.17%.^1^ The aviation industry's rapid growth also emits more pollutants, and environmental damage is increasingly severe. Compared with the domestic airlines of China and India, more international airlines are involved in international routes, which is of greater significance for discussion. For example, the airlines responsible for flying domestic routes in China are all Chinese airlines.^2^ However, international routes are different, and there are more international airlines, which is convenient for comparison (see details in Table S1. Specific steps of data collection, related to experimental procedures, Table S2. The CCD and LTO carbon emissions of China-foreign routes, related to experimental procedures, Table S3. The CCD and LTO carbon emissions of India-foreign routes, related to experimental procedures, Table S4. Specific calculation steps of carbon emission intensity, related to STAR experimental procedures, Table S5. Carbon emission intensities of each aircraft type of China-foreign routes, related to experimental procedures, Table S6. Carbon emission intensities of each aircraft type of India-foreign routes, related to experimental procedures). Therefore, studying the emissions of China-foreign and India-foreign routes is of great significance.

Carbon dioxide (CO_2_) accounts for the largest proportion of the pollutants emitted by aviation, and CO_2_ is the most important gas causing the global greenhouse effect. As a result, some studies have focused on calculating CO_2_ emissions.^3^ For example, through the International Civil Aviation Organization (ICAO) carbon emissions calculator, Larsson et al. calculated the greenhouse gas (GHG) emissions from international air travel for Sweden between 1990 and 2014.^4^ In addition, Kito et al. applied a decomposition analysis to account for the CO_2_ emissions of Japan’s two major airlines. As a result, they concluded that more Boeing 787 planes led to CO_2_ emission reductions of 1.3 million tons by the two companies.^5^ Generally, the flight process consists of seven steps: engine starting, taxiing, taking off, climbing, cruising, descending, and landing.^6^ It is usually divided into the landing and take-off (LTO) cycle and the climbing/cruising/descending (CCD) stage. To account for the gaseous pollutant emissions of aero-engine during standard LTO cycle, ICAO has successfully developed simple, advanced, and complex methods according to different calculation methods and data requirements since the 1970s.^7^ The direct use of model reference value in ICAO's simple methods will add uncertainty to the accounting results. On the other hand, the advanced methods and complex methods have highly accurate results. However, they have the limitations of high data requirements, complex implementation and high research cost and are unsuitable for mass calculation. Therefore, the development of relevant research is relatively slow. Based on the ICAO calculation system, the US Environmental Protection Agency (EPA) puts forward the EPA method combined with the actual situation.^8^ It mainly aims to calculate emission inventory during the LTO cycle. Its overall calculation idea is the same as ICAO's simple method B. Therefore, there may be no significant difference between the two calculation results for a single aircraft.

Meanwhile, the European Environment Agency (EEA) has established the European Monitoring and Evaluation Program (EMEP) cooperative action framework. The EMEP calculation method is divided into level I, II, and III methods. Level III is divided into methods A and B.^9^ It focuses on analyzing the emission characteristics of aero-engine from the fuel perspective and ignores the differences between engine types. Since then, ICAO has further improved the calculation method and proposed the ICAO carbon emissions calculator, which can estimate the aviation emission per unit passenger based on the data of various aircraft types. The ICAO carbon emissions calculator^10^ is a universal method for calculating aviation carbon emissions. The ICAO methodology uses a distance-based approach to estimate an individual's aviation emissions using data on aircraft types. The ICAO method was also used at Tbilisi International Airport in Georgia,^11^ the Hasan Polatkan airport in Turkey,^12^ and Chinese airports.^13^ However, there are some drawbacks to the method provided by ICAO. First, the distance difference is not enough. For example, according to VariFlight,^6^ A320-214 flew between 360 and 3,649 km on domestic routes in China in 2018, exceeding the methodology provided by the ICAO. Second, there is no distinction between specific aircraft. ICAO's calculation method only considers large sequences and does not consider differences between subsequences. For example, the A320 family has many families, such as the A320-100 and A320-200, with different engine types, which may lead to a significant difference in the carbon emissions of the two aircraft.^14^

As one of the aviation emission reduction measures, the fleet adjustment has also been concerned by many scholars. Alam et al. proposed an aircraft dynamic continuous descent approach path by optimizing flight procedures to put forward the methods and means of aircraft emission reduction. By prolonging the aircraft's flight time at cruise altitude and reducing the flight in the level flight section, the pollutant emissions of the aircraft can be reduced in two dimensions: horizontal and vertical sections.^15^ The theoretical and practical significance of the continuous descent approach in reducing aviation emission pollution for Louisville International Airport has been evaluated based on system analysis, considering the economic benefits of aviation operation and aircraft exhaust emission.^16^ The optimal vertical trajectory of the aircraft and numerically simulated different models of B737-500 and B767-400 were generated, verifying the advantages of this method in shortening flight time, saving fuel consumption, and reducing airport noise and aircraft emissions.^17^ Furthermore, flight tests on B757 aircraft verified that the continuous descent approach saves 50–150 kg fuel and reduces 150–450 kg exhaust emissions compared with the step approach.^18^

By summarizing the existing literature, we can conclude that the simultaneous accounting of multiple pollutants cannot be realized, and finer sub-models and broader distance segments are not considered. Emission reduction measures mainly optimize the aircraft's flight procedures and trajectories. From the perspective of airlines, there is still a lack of research on the impact of aircraft-type replacement on aviation emission reduction. The critical mitigation measures for the aviation industry include using biomass fuels, airline fleet updates, and emission market mechanisms.^19^ Furthermore, as a vital part of an airline's fleet upgrade, fleet schedule reconfiguration is a part of the airline's day-to-day operations. For example, according to the Civil Aviation Administration of China (CAAC), in the actual process of flights, many factors may cause airlines to temporarily adjust their aircraft, such as flight delay, aircraft failure, fuel cost, emergencies, etc. Therefore, the CAAC allows airlines to declare multiple aircraft in the flight plan. In addition, after the “3.21” 737-800 passenger plane crash, China Eastern Airlines temporarily grounded all 737-800 and replaced the route with the A320 series.^20^ Therefore, fleet schedule reconfiguration is very common. In this paper, we apply the modified fuel percentage method (MFPM)^14^ to calculate the carbon emissions of the CCD stage and the ICAO standard method to get the emissions of the LTO stage. We divide each route into multiple distance segments every 500 km, then calculate each aircraft's emission intensity in each distance segment to get the aircraft with the lowest emission intensity in each distance segment (see details in Tables S3 and S4). Then, we use this aircraft to replace all aircraft at the same distance, recalculate the emissions, and analyze the changes in emissions. It should be noted that this article considered the fleet of airlines in 2019. If the aircraft with the lowest emission intensity is not in the airline’s fleet, the aircraft with the second-lowest emission intensity will be considered, and so on.

## Results

Since COVID-19 began to rage around the world in 2020, the data for 2020 and 2021 cannot be used as a reference. Therefore, this paper selects the data of China-foreign and India-foreign routes in 2019 as the research object.

### Statistical characteristics of China-foreign and India-foreign routes and the accuracy of the method

**Figure 1:**
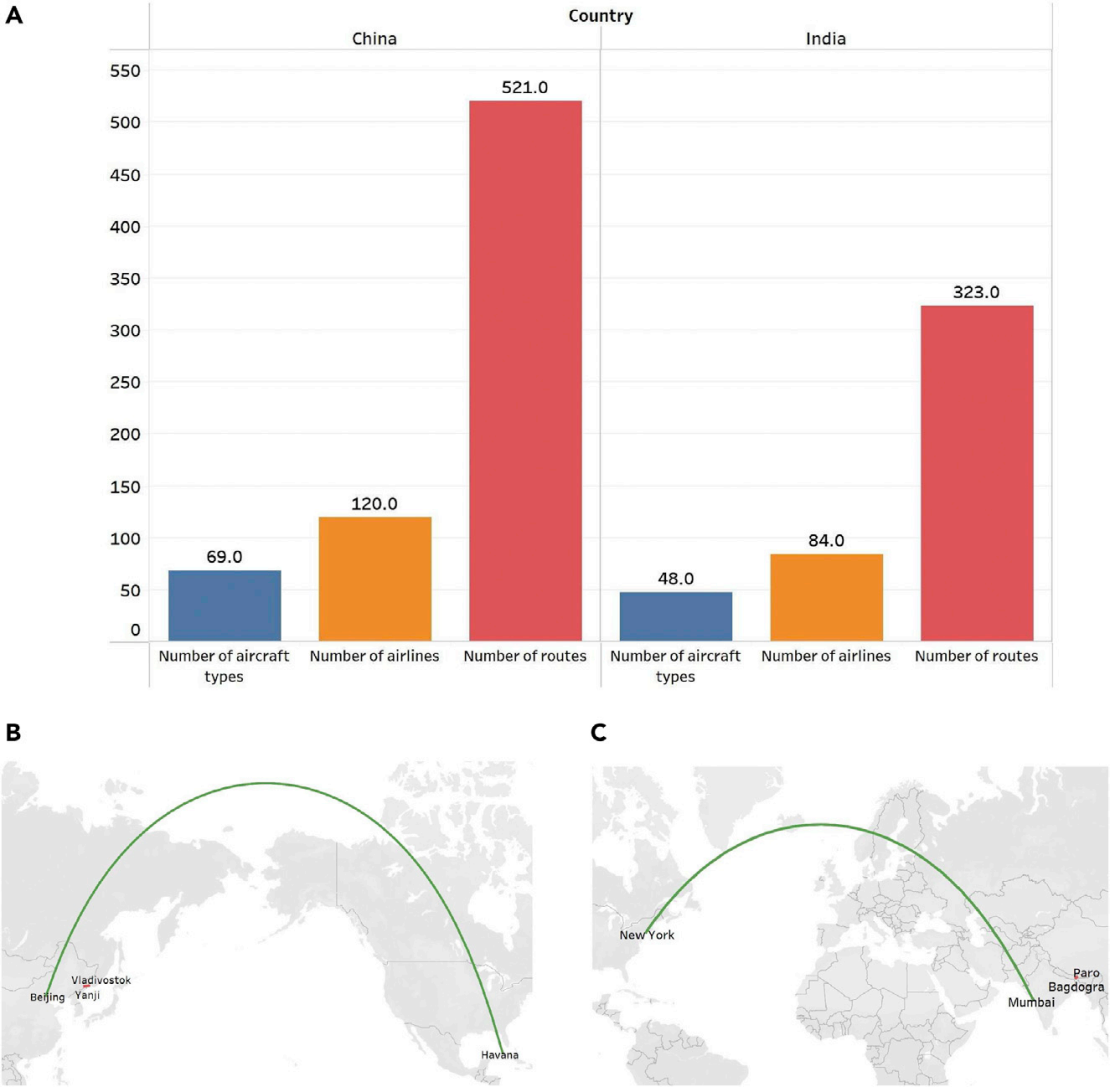
Statistical characters of the routes in 2019 (A) The basic information. (B) The farthest and nearest distances of China-foreign routes. (C) The farthest and nearest distances of India-foreign routes.

This article collects information on China-foreign routes and India-foreign routes from 2019, and the detailed statistical features are shown in Figure 1. Figure 1A shows that the China-foreign routes in 2019 involved 69 aircraft types, and there were 521 routes and 120 airlines. These routes cover 62 countries, indicating that China has fixed flights globally with more than 60 countries. Figure 1B shows that the farthest route in 2019 was Beijing-Havana (13,091 km). From this result, in 2019, the most distant place between China and foreign routes was North America. The nearest route was Yanji-Vladivostok (224 km). In contrast, in 2019, the India-foreign exchange frequency was about 60% of the China-foreign exchange frequency. The routes between India and foreign countries in 2019 involved 48 types of aircraft, 323 routes, and 84 airlines. These routes covered 52 countries. Figure 1C shows that the farthest route in 2019 was Mumbai-New York (12,565 km). From this result, in 2019, North America was the most distant place on India’s external routes. The nearest route was Bagdogra-Paro (135 km).

Then, we will discuss the accuracy of the method. According to the data released by the CAAC, in 2019, 46.374 billion tons-km of transportation was completed between China and foreign routes.^21^ The fuel consumption per turnover of China's domestic and foreign routes is about 0.285 kg/ton-km, but the international routes can float 5% due to the large aircraft used. The fuel consumption of unit turnover is 0.2993 kg/ton-km. Using this standard, multiplied by the carbon emission coefficient per fuel consumption (3.157 kg/kg), the CO_2_ emissions of the China-foreign routes in 2019 would be 43,818,333.50 tons. Therefore, the CO_2_ emissions calculated in this paper are 45,021,648.34 tons, with a 2.75% error rate. Similarly, the error in India-foreign routes in 2019 was 5.21%. Considering that the statistical data of various airlines may also have errors, the calculation results of this paper are very accurate.

### The impacts of fleet schedule reconfiguration on overall carbon emissions

In this part, we compared CO_2_ changes in overall emissions, average emissions of routes, and average emissions of airlines before and after fleet schedule reconfiguration. In this paper, we segment each route according to a segment of 500 km. Therefore, the China-foreign routes in 2019 are divided into 27 distance segments: 0–500; 501–1,000; 1,001–1,500; 1,501–2,000; 2,001–2,500; 2,501–3,000; 3,001–3,500; 3,501–4,000; 4,001–4,500; 4,501–5,000; 5,001–5,500; 5,501–6,000; 6,001–6,500; 6,501–7,000; 7,001–7,500, 7,501–8,000; 8,001–8,500; 8,501–9,000; 9,001–9,500; 9,501–10,000; 10,001–10,500; 10,501–11,000; 11,001–11,500; 11,501–12,000; 12,001–12,500; 12,501–13,000; and 13,001–13,500 km. The India-foreign routes in 2019 are divided into 22 distance segments: 0–500; 501–1,000; 1,001–1,500; 1,501–2,000; 2,001–2,500; 2,501–3,000; 3,001–3,500; 3,501–4,000; 4,001–4,500; 4,501–5,000; 5,001–5,500; 5,501–6,000; 6,001–6,500; 6,501–7,000; 7,001–7,500; 7,501–8,000; 8,001–8,500; 10,001–10,500; 11,001–11,500; 11,501–12,000; 12,001–12,500; and 12,501–13,000 km. The primary emissions include CO_2_, CO, HC, NOx, SO_2_, and PM2.5. We calculate the carbon emission intensity in CCD stage according to the distance segments (see details in Tables S5 and S6).

**Figure 2:**
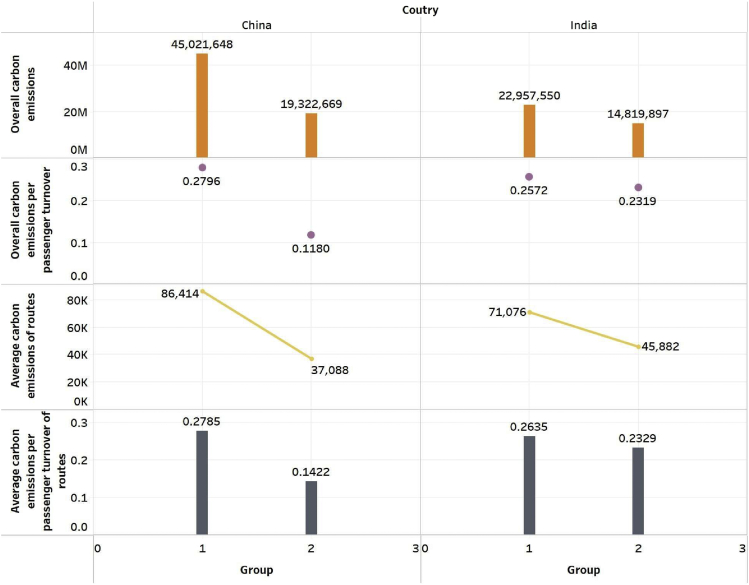
The changes of the carbon emissions before and after reconfiguration

We select the aircraft type with the lowest emission intensity in the CCD stage in each distance segment as the optimal configuration based on these distance segments. If the aircraft with the lowest emission intensity is not in the airline’s fleet, the aircraft with the second-lowest emission intensity will be considered. Then, we compare the two data groups before and after the fleet schedule reconfiguration. Figure 2 shows the changes in the overall carbon emissions in the two groups of data (group 1 represents original data and group 2 optimized data). After the fleet schedule reconfiguration, the carbon emissions of the China-foreign routes and the India-foreign routes in 2019 were reduced. The overall carbon emissions of China-foreign routes have decreased more than that of India-foreign routes. The overall emissions of CO_2_ of China-foreign routes decreased by 57.08%, and the India-foreign routes decreased by 35.45%.

Then, we calculate the changes in carbon emission costs and aircraft costs before and after fleet schedule reconfiguration to reflect the critical impact of aircraft optimization on routes and airlines. First, the carbon prices of various regions differed in 2019, but the difference is not significant.^22^ The average daily trading price of the EU carbon market fluctuated between $29.45 and $31.66/ton; the carbon market price in South Korea was stable at $24.00–$24.13/ton; the carbon market price in New Zealand fluctuated between $22.90 and $23.22/ton; and the North American carbon market was about $15.73–$17.45/ton. Therefore, the average carbon price was $23.57/ton. And for the changes in aircraft costs before and after fleet schedule reconfiguration. This paper calculates the changes in aircraft costs before and after the reconfiguration according to the aircraft price.

**Figure 3:**
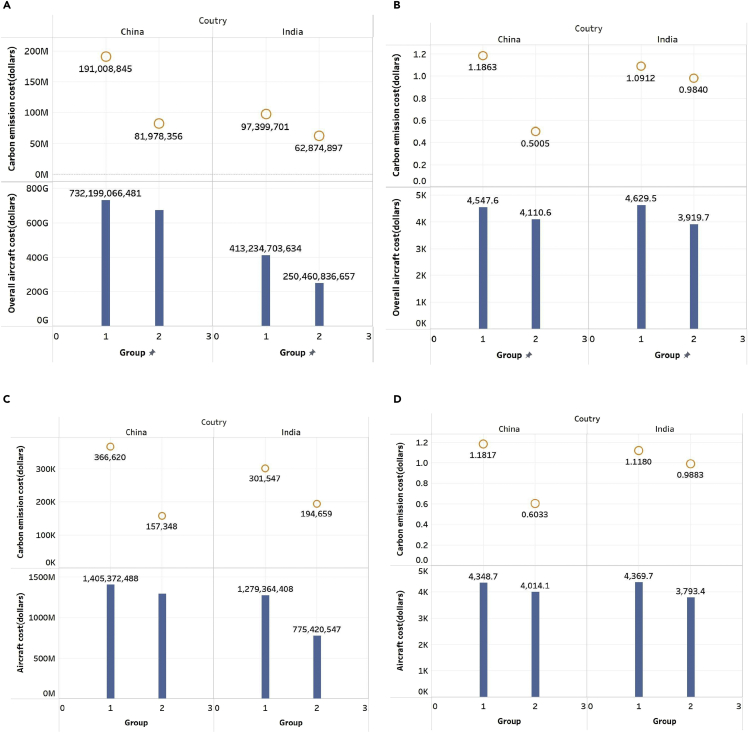
The changes of carbon emission costs and overall aircraft costs before and after fleet schedule reconfiguration (dollars) (A) Overall carbon emission costs and overall aircraft costs before and after fleet schedule reconfiguration (dollars). (B) Overall carbon emission costs per passenger turnover and aircraft costs per passenger turnover. (C) Average carbon emission costs and aircraft costs of routes (dollars). (D) Average carbon emission costs per passenger turnover and aircraft costs per passenger turnover (dollars).

Figure 3 shows the changes in carbon emission costs and overall aircraft costs caused by the change of fleet schedule reconfiguration of China-foreign routes and India-foreign routes in 2019 (group 1 represents original data and group 2 optimized data). As shown in Figure 3A, the costs of both China-foreign and India-foreign routes have declined after fleet schedule reconfiguration. China-foreign routes’ overall carbon emission costs decrease by 57.08%, and the overall aircraft costs decrease by 8.04%. The overall carbon emission costs of India-foreign routes decrease by 35.45%, and the overall aircraft costs decrease by 39.39%.

### The impacts of fleet schedule reconfiguration on the carbon emissions per passenger turnover

Further, compare the carbon emissions per passenger turnover changes before and after fleet schedule reconfiguration. Figure 2 shows the carbon emissions per passenger turnover before and after the fleet schedule reconfiguration on China-foreign and India-foreign routes. Like the change in overall emissions, after fleet schedule reconfiguration, the carbon emissions per passenger turnover of the China-foreign and the India-foreign routes in 2019 have been reduced, and the emissions per passenger turnover of China-foreign routes have decreased more than that of India-foreign routes. The emissions per passenger turnover of CO_2_ of China-foreign routes decreased by 57.81%, and the India-foreign routes decreased by 9.82%.

Then, Figure 3B shows the changes in carbon emission costs per passenger turnover and aircraft costs per passenger turnover caused by the change of fleet schedule reconfiguration of China-foreign routes and India-foreign routes in 2019. Similarly, the carbon emission cost per passenger turnover also considers the reduction ratio of 82%. As shown in Figure 3B, the costs of both China-foreign and India-foreign routes have decreased after reconfiguring the fleet schedule. As a result, the carbon emission costs per passenger turnover of China-foreign routes decreased by $0.69 (57.81%), and the aircraft costs per passenger decreased by $437.04 (9.61%). On the other hand, the carbon emission costs per passenger turnover of India-foreign routes decreased by $0.11 (9.82%), and the aircraft costs per passenger decreased by $709.79 (15.33%).

### The impacts of fleet schedule reconfiguration on the routes

Figure 2 shows the changes in the average carbon emissions of routes before and after fleet schedule reconfiguration. The decline rate of CO_2_ is the same as that of their overall emissions. Figure 3C shows the changes in the average carbon emission costs and aircraft costs of routes before and after fleet schedule reconfiguration. The average carbon emission and aircraft costs of China-foreign and India-foreign routes decreased after fleet schedule reconfiguration. China-foreign routes’ average carbon emission costs decrease by 209,271.57 dollars, and the average aircraft costs decrease by $113,013,684.15. The decline of India-foreign routes is also significant. The average carbon emission cost decreased by $106,887.94, and the average aircraft costs decreased by $503,943,860.61.

**Figure 4:**
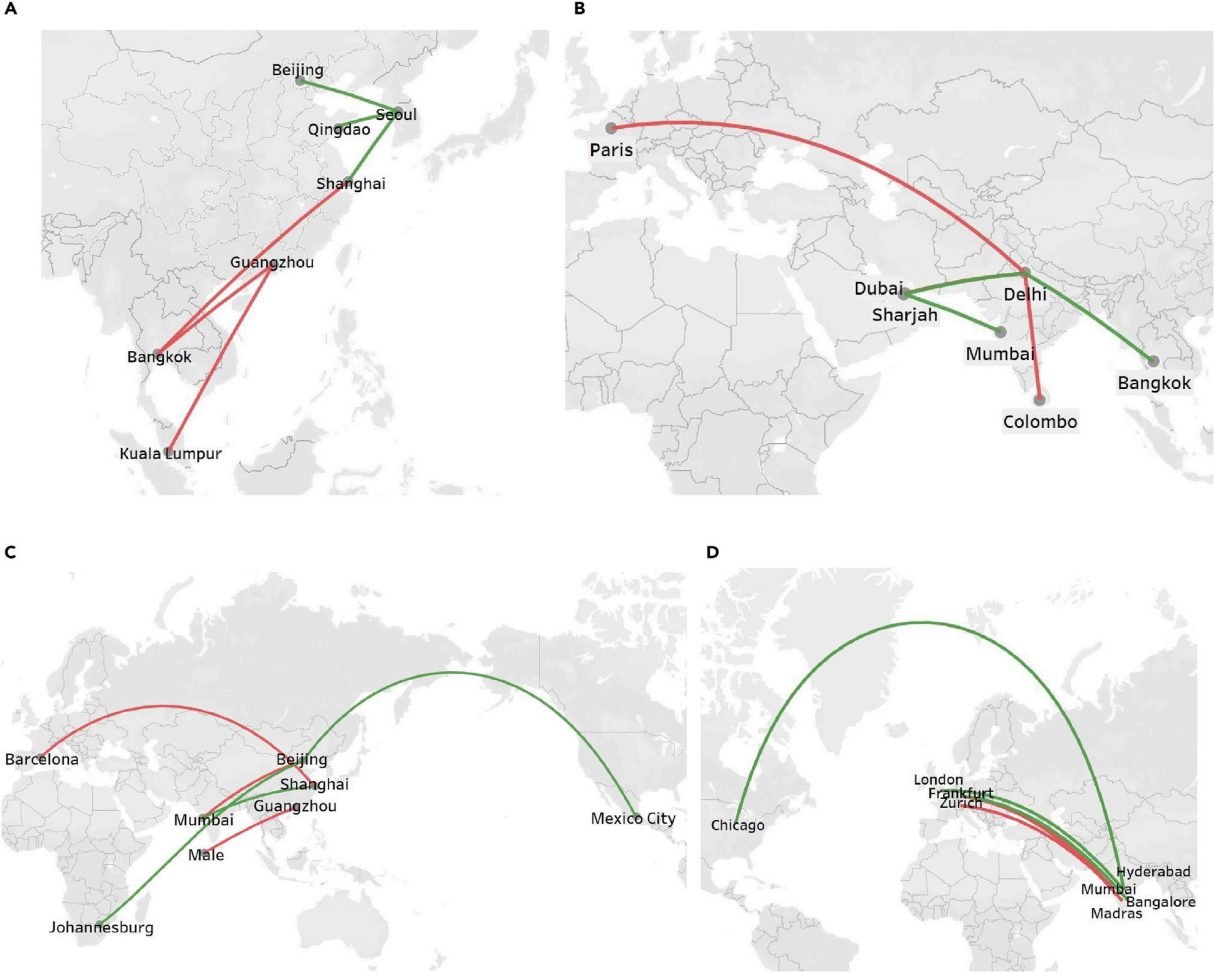
The impacts of aircraft reorganization on carbon emission costs and aircraft costs of routes (A and B) The three routes with the largest reduction and the three routes with the largest increase of (A) China-foreign and (B) India-foreign routes in 2019. (C and D) The three routes with the largest reduction and the three routes with the largest increase of (C) China-foreign and (D) India-foreign routes for unit passenger turnover costs in 2019.

In addition, we compare the carbon emission costs and aircraft costs of China-foreign routes and India-foreign routes before and after fleet schedule reconfiguration in 2019 and study the impact of the fleet schedule reconfiguration on them. Finally, we add the changes in carbon emission costs and aircraft costs after fleet schedule reconfiguration on routes to obtain the overall difference in the costs of routes. Figure 4A presents the three routes with the most significant reduction and the three routes with the largest increase of China-foreign routes in 2019. The red is the most marked increase, and the green is the route with the most considerable reduction. Among the China-foreign routes, Seoul-Shanghai, Seoul-Beijing, and Seoul-Qingdao are the three most significant cost-reduction routes. The distance of these routes is less than 1,000 km, showing that Shanghai and Beijing are two major aviation hubs in China. Therefore, the optimal aircraft allocation will positively impact their routes’ economic benefits. However, the optimal aircraft allocation may have a particularly negative impact from the perspective of the three routes with the most significant costs increase (Kuala Lumpur-Guangzhou, Bangkok-Shanghai, and Bangkok-Guangzhou) on the economic benefits of routes in Guangzhou, another major aviation city in China. And the distance of these routes is more significant than 1,500 km.

Similarly, Figure 4B presents the three routes with the most significant reduction and the three routes with the largest increase of India-foreign routes in 2019. The three routes with the most effective cost reduction of India-foreign routes in 2019 are Mumbai-Dubai, Delhi-Dubai, and Delhi-Bangkok. Delhi-Sharjah, Delhi-Paris, and Delhi-Colombo are the three routes with the most significant cost increase for India-foreign routes in 2019. It is worth noting that for both China-foreign and India-foreign routes, the carbon emission costs of their routes will decrease after fleet schedule reconfiguration. Therefore, the changes in total costs mainly depend on aircraft costs.

Figure 2 shows that this paper compares the average carbon emissions per passenger of routes before and after fleet reconfiguration of China-foreign and India-foreign routes in 2019. After reconfiguring the aircraft, the average carbon emissions per passenger turnover of the China-foreign and the India-foreign routes in 2019 have been reduced. The average carbon emissions per passenger turnover of China-foreign routes decreased by 48.95%. The India-foreign routes decreased by 11.60%. In 2019, among the 521 China-foreign routes, 131 routes’ carbon emissions per passenger turnover increased after fleet schedule reconfiguration, 19 routes did not change, and the remaining 371 routes’ carbon emissions per passenger turnover decreased. The five routes with the most significant increase in carbon emissions per passenger turnover of China-foreign routes are Seoul-Dalian, Hanoi-Nanning, Seoul-Qingdao, Seoul-Weihai, and Seoul-Yantai. The distance of these five routes is between 0 and 500 km. The aircraft type of the 0–500 segment of China-foreign routes is optimized and adjusted to DHC-8-402, with a passenger capacity of only 74 people. Therefore, the carbon emission per passenger turnover has increased significantly.

Similarly, among the 323 routes between India and foreign countries in 2019, the carbon emissions per passenger turnover of 75 routes increased after fleet schedule reconfiguration, 15 routes did not change, and the carbon emissions per passenger turnover of the remaining 233 routes decreased. The five routes with the most significant increase in unit passenger turnover of India-foreign routes are Bangalore-Frankfurt, Mumbai-Paris, Mumbai-Teheran, Trivandrum-Colombo, and Tiruchirapally-Colombo. Among them, the carbon emissions per passenger turnover of Trivandrum-Colombo and Tiruchirapally-Colombo increased by more than 40% after fleet schedule reconfiguration. The distance between Trivandrum-Colombo and Tiruchirapally-Colombo is between 0 and 500 km. The aircraft type of the 0–500 segment of India-foreign routes is optimized and adjusted to ATR (avions de transport regional), with a passenger capacity of only 69 people.

Figure 3D presents the changes in average carbon emission costs per passenger turnover and aircraft costs per passenger turnover of routes before and after fleet schedule reconfiguration (group 1 represents original data and group 2 optimized data). Similarly, China-foreign and India-foreign routes’ average carbon emission costs per passenger and aircraft costs per passenger turnover decreased after reconfiguration. The average carbon emission costs per passenger turnover of China-foreign routes decreased by $0.5785, and the average aircraft costs per passenger turnover decreased by $334.60. The decline of India-foreign routes is also significant. The average carbon emission costs per passenger turnover decreased by $0.1297, and the average aircraft costs decreased by $576.21.

Figure 4 also presents the impacts of aircraft reorganization on carbon emission costs per passenger turnover and aircraft costs per passenger turnover of routes. Figure 4C shows that the three routes with the most considerable costs per passenger turnover reduction among the China-foreign routes are Johannesburg-Beijing, Mexico City-Beijing, and Mumbai-Shanghai. Mumbai-Beijing, Male-Guangzhou, and Barcelona-Shanghai are the three routes with the highest cost-per-passenger turnover increase. Similarly, Figure 4D presents the three routes with the most significant reduction and the three routes with the largest expansion of India-foreign routes in 2019. The three routes with the most extensive costs per passenger reduction of India-foreign routes in 2019 are Hyderabad-Chicago, Madras-Frankfurt, and Hyderabad-London. Mumbai-Frankfurt, Bangalore-Frankfurt, and Mumbai-Zurich have the highest costs per passenger increase of India-foreign routes in 2019. It can be found that most of the cost reduction of unit passenger turnover of China-foreign routes and India-foreign routes are long-distance routes (>5,000 km).

### The impacts of fleet schedule reconfiguration on airlines

Table 1 shows the changes in the average carbon emissions of airlines before and after fleet schedule reconfiguration. The decline rate is also the same as that of their overall emissions. This shows that the optimal configuration of aircraft is also highly effective in controlling pollutant emissions for airlines. Table 1 also shows the changes in airlines’ average carbon emission costs and aircraft costs before and after fleet schedule reconfiguration. From the perspective of airlines, after the fleet schedule reconfiguration, the average carbon emission costs and aircraft costs of both China-foreign and India-foreign airlines have decreased. China-foreign airlines’ average carbon emission costs decreased by $908,587.41, and the average aircraft costs decreased by $491,588,848.03. The decline of India-foreign routes is also significant. The average carbon emission cost decreased by $411,009.57, and the average aircraft costs decreased by $1,937,784,130.68. In other words, the average economic benefit of airlines increases after fleet schedule reconfiguration.

**Table 1:** Comparison of carbon emissions, carbon emission costs, and aircraft costs of the airlines before and after fleet schedule reconfiguration

Items		China-foreign routes			India-foreign routes		
		Original	Optimized	Difference	Original	Optimized	Difference
CO_2_	AEs (tons)	375,180.4	161,022.2	−214,158	273,304.2	176,427.3	−96,876.9
	AEPT (tons/person-km)	2.81E−01	1.28E−01	−0.153	2.64E−01	2.32E−01	−0.032
Carbon emission costs	AEs (dollars)	1,591,740.4	683,152.96	−908,587	1,159,520.2	748,510.67	−411,010
	AEPT (dollars/person-km)	1.1912612	0.5416531	−0.64961	1.1187733	0.9861037	−0.13267
Aircraft costs	AEs (dollars)	6.092E+09	5.601E+09	−4.9E+08	4.919E+09	2.982E+09	−1.9E+09
	AEPT (dollars/person-km)	4,531.6624	4,103.9207	−427.742	4,403.0042	3,803.4403	−599.564

AEs, average emissions; AEPT, average emissions per passenger turnover.

**Figure 5:**
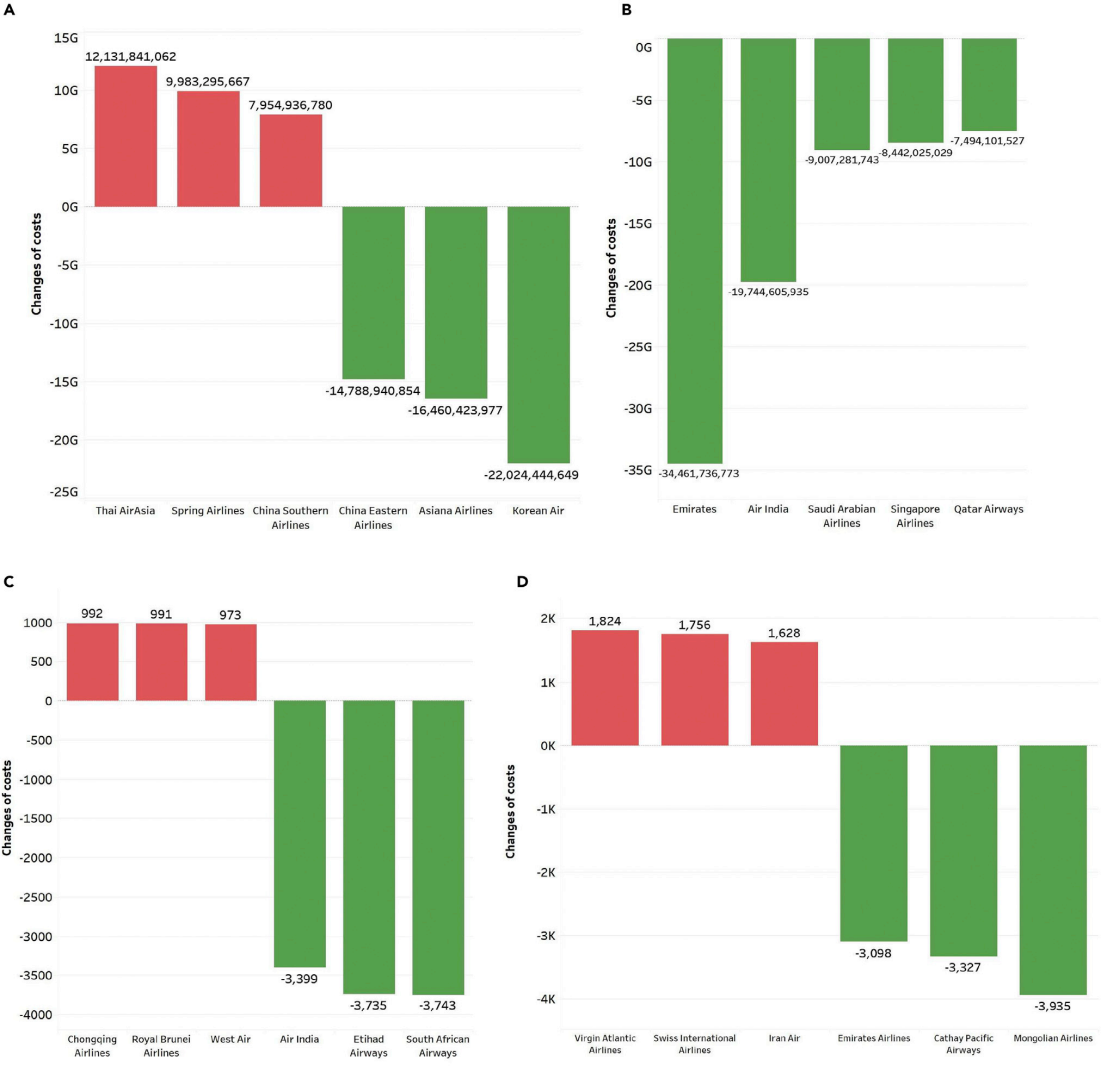
The impacts of aircraft reorganization on carbon emission costs and aircraft costs of airlines (A) The three airlines with the largest reduction and the three airlines with the largest increase of China-foreign airlines in 2019. (B) The five airlines with the largest reduction of India-foreign airlines in 2019. (C) The three airlines with the largest reduction and the three airlines with the largest increase of China-foreign airlines for unit passenger turnover costs in 2019. (D) The three airlines with the largest reduction and the three airlines with the largest increase of India-foreign airlines for unit passenger turnover costs in 2019.

Then, we compare airlines’ carbon emissions and aircraft costs before and after fleet reconfiguration in 2019. Similarly, we add the changes in carbon emission costs and aircraft costs after fleet schedule reconfiguration to obtain the overall cost change of airlines. Figure 5A presents the three airlines with the most considerable reduction and the most significant increase in China-foreign routes in 2019. The red is the airline with the most marked increase, and the green is the airline with the most considerable reduction. Among the China-foreign airlines, the three airlines with the most notable cost reduction are Korean Air, Asiana Airlines, and China Eastern Airlines, and the three airlines with the most significant cost increase are China Southern Airline, Spring Airlines, and Thai AirAsia. The main reason is that the aircraft cost changes significantly. Therefore, different from airlines on China-foreign routes, the cost change of all airlines on India-foreign routes declined after the fleet schedule reconfiguration. Except for five airlines, the costs have not changed, and the carbon emission and aircraft costs of the remaining 79 airlines on India-foreign routes have decreased. Figure 5B presents the five airlines with the most considerable reduction of India-foreign airlines in 2019: Emirates, Air India, Saudi Arabian Airlines, Singapore Airlines, and Qatar Airways.

As shown in Table 1, further compare the average carbon emissions per passenger of airlines before and after the fleet schedule reconfiguration of China-foreign airlines and India-foreign airlines in 2019. After reconfiguring the aircraft, airlines’ average carbon emissions per passenger turnover of the China-foreign airlines and the India-foreign airlines in 2019 have dropped. Airlines’ average carbon emissions per passenger turnover of China-foreign airlines decreased by 54.53%. The India-foreign airlines decreased by 11.86%. Table 1 also presents the changes in average carbon emission costs per passenger turnover and airline costs per passenger turnover before and after fleet reconfiguration. Similarly, the average carbon emission costs per passenger turnover and aircraft costs per passenger turnover of both China-foreign and India-foreign airlines decreased after reconfiguration. The average carbon emission costs per passenger turnover of China-foreign airlines decreased by $0.6496, and the average aircraft costs per passenger turnover decreased by $427.74. The decline of India-foreign airlines is also significant. The average carbon emission costs per passenger turnover decreased by $0.1327, and the average aircraft costs decreased by $599.56.

Figure 5 also presents the impacts of aircraft reorganization on carbon emission costs per passenger turnover and aircraft costs per passenger turnover. Figure 5C shows that the three airlines with the most considerable costs per passenger turnover reduction are South African Airways, Etihad Airways, and Air India, among China-foreign routes. West Air, Royal Brunei Airlines, and Chongqing Airlines are three airlines with enormous costs per passenger turnover increase. And Figure 5D presents the three airlines with the most significant reduction and the three airlines with the largest increase of India-foreign routes in 2019. The three airlines with the most extensive costs per passenger reduction of India-foreign airlines in 2019 are Mongolian Airlines, Cathay Pacific Airways, and Emirates Airlines. On the other hand, Iran Air, Swiss International Airlines, and Virgin Atlantic Airlines have the highest costs per passenger increase on India-foreign routes in 2019. It can be found that although the aircraft costs on India-foreign routes are decreasing, the sum of the carbon emission costs per passenger turnover and aircraft costs per passenger turnover of airlines may increase.

Finally, we analyze those airlines whose carbon emission costs have decreased but their aircraft costs have increased. In this regard, there are significant differences between India's and China's foreign routes. After aircraft configuration, all airlines have reduced their carbon emissions and aircraft costs for India's external routes. Still, among the 120 airlines on China's external routes, 58 airlines have reduced their carbon emissions, but aircraft costs have increased. We analyzed these 58 airlines and found that when the carbon emission price increases, the part of carbon emission cost decrease can offset the portion of aircraft cost increase. However, the results show that these two costs can be compensated only when the carbon price is 8.2 times higher than the current carbon price. The most significant multiple is Myanmar Airlines, by about 27.48 times, and the smallest is Garuda Indonesia, only 0.079 times. This shows that Myanmar Airlines’ rising aircraft costs are far greater than the falling part of carbon emission reduction costs.

## Discussion

The focus of this study is to explore the impact of fleet schedule reconfiguration on aircraft carbon emissions from China-foreign and India-foreign routes in 2019 and to calculate the changes in carbon emission costs and aircraft costs. First, we collect the flight information of all international routes between China, India, and foreign countries (including aircraft types, flight frequency, airline, flying distance, flying time, etc.). Then, we calculate the overall carbon emissions of each route and airline. The overall emissions include CCD emissions and LTO emissions. CCD emissions are calculated by the MFPM, and LTO emissions are calculated based on the ICAO standard method. Then, we reconfigure the aircraft types of each route according to the carbon emission intensity and calculate its impact on carbon emissions, carbon emission costs, aircraft costs, and so on. As a result, accounting can better summarize the critical implications of the optimal aircraft configuration in aviation and provide data and method references for implementing aviation emission reduction strategies. The results show that aircraft configuration is a feasible way of emissions reduction for the whole of or most airlines.

The main contribution of this paper to the literature is reflected in the following aspects. This is the first attempt to analyze the impact of fleet schedule reconfiguration on aircraft carbon emissions. Many studies focus on the efficiency of airlines, but few previous studies have focused on the effects of aircraft replacement on aviation emissions and costs. Our work can fill this gap. We divide the route distance into several groups and get the carbon emissions of aircraft types at these different distances to ensure that the calculation results are accurate. Then, we get the aircraft with the lowest carbon emission intensity in each distance segment and use it to replace the original aircraft to discuss the situation of emission reduction. In addition, we calculated the cost savings due to carbon emission reduction. We also discussed the cost increase after reconfiguring aircraft to analyze which carbon price can make emission reduction cost savings for some airlines to offset the rise in aircraft costs. Furthermore, this paper calculates the carbon emissions from China’s and India’s external routes, enriching the existing database. In general, with the advent of a low-carbon economy, realizing the goal of a zero-carbon vision in the air transport industry requires that the entire air transport industry and the country face it actively. This paper provides a new database for air carbon emission calculations, a new path for air carbon emission reduction, and a reference for countries to explore sustainable development strategies and develop a low-carbon economy in the air transport industry. And the research results provide a connection for governments to explore sustainable development strategies and develop a low-carbon economy in the air transport industry.

The main conclusions and policy recommendations are as follows: first, after the reconfiguration of the aircraft types, the overall carbon emissions of the China-foreign routes and the India-foreign routes in 2019 have been reduced, and the overall carbon emissions of China-foreign routes have decreased more than that of India-foreign routes. The overall CO_2_ emissions of China-foreign routes decreased by 57.08%, and the India-foreign routes decreased by 35.45%. Second, in terms of overall cost changes, the costs of carbon emission on China-foreign routes decreased by 57.08%, and the overall costs of aircraft decreased by 8.04%. The overall carbon emission costs of Indian-foreign routes and the overall costs of aircraft decreased by 35.45% and 39.39%, respectively. Third, like the change in overall emissions, after reconfiguring the aircraft types, the carbon emissions per passenger turnover of the China-foreign and the India-foreign routes in 2019 have declined. The carbon emissions per passenger turnover of China-foreign and India-foreign routes decreased by 57.81% and 9.82%, respectively. Fourth, analyzing the emissions and costs of routes and airlines, the decline rate of the routes and airlines of CO_2_ is the same as that of the overall carbon emissions. Fifth, it is worth noting that for both China-foreign and India-foreign routes, the carbon emission costs of their routes will decrease after the reconfiguration of aircraft types. Therefore, the changes in total costs mainly depend on aircraft costs. For the changes in unit passenger turnover costs of specific routes, it can be found that most of the cost reduction of unit passenger turnover of China-foreign and India-foreign routes are long-distance routes (>7,000 km). Sixth, for the specific airlines Air China and China Eastern Airlines, the top two airlines in China, we show that the optimal configuration of aircraft types will significantly impact them. Unlike airlines on China-foreign routes, the sum of cost changes of all airlines on India-foreign routes has declined. The reason is that after the aircraft-type reconfiguration, except for five airlines, the costs have not changed, and the carbon emission and aircraft costs of the remaining 79 airlines on India-foreign routes have decreased. And although airlines’ costs on India-foreign routes are decreasing, the sum of the carbon emission costs per passenger turnover and aircraft costs per passenger turnover of airlines may increase.

### Limitations of the study

It should be noted that the overall emissions are calculated through the standard LTO stage. Therefore, emissions due to delays are not considered, and aircraft transfer caused by temporary weather is not considered. Furthermore, this study has not evaluated emissions from air cargo. Therefore, further investigation can calculate the emissions caused by delay, aircraft transfer, and air cargo.

## Experimental procedures

### Resource availability

#### Lead contact

Further information and requests should be directed to the lead author, Qiang Cui (cuiqiang@seu.edu.cn).

#### Materials availability

This study did not generate new unique materials.

### Method details

#### The MFPM

In the MFPM method, the CCD emissions E(Q) can be calculated by $E_{j}\left(Q\right)=I_{j}\times F\left(Q\right)=I_{j}\times M_{fuel}\times weight\left(Q\right)=I_{j}\times \left(1-M_{ff}\right)\times weight\left(Q\right)$ = $I_{j}\times \left(1-\prod_{i=1}^{n}\frac{W_{i}}{W_{i-1}}\right)\times weight\left(Q\right)=I_{j}\times \left[1-e^{-\frac{dis\times ratio_{cr}}{10\times v}}\right]\times weight\left(Q\right)$ = $I_{j}\times \left[1-e^{-\frac{dis\times ratio_{cr}}{10\times v}}\right]\times \left(aircraftbareweight+100\times \left(load\;factor\times number\;of\;seats\right)+50\times seat\right)$.

$I_{j}$ is the emission coefficient of pollution *j* of aviation kerosene.^23^ weight(Q) is the total weight of the aircraft. $M_{fuel}$ is the fuel coefficient, and $M_{ff}=\prod_{i=1}^{n}\frac{W_{i}}{W_{i-1}}\;$ is a fuel weight proportionality coefficient, which is usually calculated by FPM. The total sections of a whole flight contain seven task sections: engine starting, taxiing, taking off, climbing, cruising, descending, and landing. $\frac{W_{i}}{W_{i-1}}$ is the fuel weight proportionality coefficient of task section i(i=1,2,…,7). numberofseats is the certified seat number, and seat is the actual passenger number.

As we only consider the CCD section in this study, we define the $\frac{W_{i}}{W_{i-1}}$ of other sections as 1. The $\frac{W_{i}}{W_{i-1}}$ of climbing and descending are 0.980 and 0.990, respectively. The equation of the CCD section to calculate $\frac{W_{i}}{W_{i-1}}$ is $\frac{W_{i}}{W_{i-1}}=e^{-\frac{dis\times c_{cr}}{10\times v\times LD_{cr}}}$. dis is the cruising distance, v is the cruising speed, $c_{cr}$ is the fuel consumption ratio when the aircraft is cruising, and $LD_{cr}$ is the lift-drag ratio when the aircraft is cruising. The value of $c_{cr}$ and $LD_{cr}$ has direct relationships with the aircraft type. We define $ratio_{cr}=\frac{c_{cr}}{LDc_{cr}}$, and then for the cruising task section, the $\frac{W_{i}}{W_{i-1}}$ is $\frac{W_{i}}{W_{i-1}}=e^{-\frac{dis\times ratio_{cr}}{10\times v}}$.

The actual flying time of each flight is applied to check the results of $ratio_{cr}$ and get the emission intensity.

For CO_2_, the emission coefficient is fixed, which is *I*_*CO*2_ = 3.157 kg/kg.

#### ICAO standard method

The LTO stage refers to the aircraft's whole process during takeoff and landing. This paper uses the standard LTO cycle definition specified by ICAO to calculate the fuel consumption, including all activities at an altitude below 3,000 feet (915 m) near the airport. Therefore, this stage is not directly related to the route. In addition, the climbing process requires higher fuel consumption than the cruise phase at a constant altitude. Thus, the CCD cycle is defined as all activities that occur at a height of 3,000 feet (915 meters). Thus, fuel use accounts for most of the whole voyage and is directly related to the flight distance.

The calculation formula of the five non-CO_2_ pollution emissions in the LTO stage is $E_{LTO}=\sum_{m}P_{a}\times N_{a}\times C_{m}\times t_{m}$. $E_{LTO}$ is the emissions in the LTO stage, $P_{a}$ is the standard emissions of the engine of aircraft type *a* (unit: kg), $N_{a}$ is the number of engines of aircraft type *a*, $C_{m}$ is the thrust setting of stage *m*, and $t_{m}$ is the working time of phase *m*. The value range of *m* is 1, 2, 3, and 4, respectively corresponding to the four stages of takeoff and landing in the aircraft flight process: takeoff, climb, approach, and taxiing. According to the standard LTO cycles defined by ICAO, when the aircraft is taking off, its engines are at 100% thrust, and working time is 0.7 min; when the aircraft is climbing, its engines are at 85% thrust, and working time is 2.2 min; when the aircraft is approaching, its engines are at 30% thrust, and working time is 4 min; and when the aircraft is taxiing, its engines are at 7% thrust, and working time is 26 min. Therefore, in a standard LTO cycle, the total working time is 32.9 min.

The fuel consumption rate is calculated as $F_{am}=\frac{1}{A}\sum_{j}K_{j}F_{jm}$. A is the total number of airlines with aircraft type *a*, *j* is the type of engine of the aircraft, $K_{j}$ is the number of aircraft type *a* equipped with engine type *j*, and $F_{jm}$ is the fuel consumption rate of engine type *j* under the *m* setting. The data are from the ICAO Aircraft Engine Emissions Databank.^23^ This formula is based on the weighted average of all possible engine types of the domestic routes in China. The data on aircraft type, flying time, flying distance, transfer flight, and airlines are from VariFlight.com.^6^ The information on the engines of each aircraft and the data on the engines are from the ICAO Aircraft Engine Emissions Databank.^23^ The information on aircraft price is from the AeroChina website.^24^

## Data Availability

This paper analyzes existing, publicly available data. The data sources and specific steps for data collection are in Table S1. The CCD and LTO carbon emissions of each route and airline are shown in Tables S2 (China-foreign routes) and S3 (India-foreign routes). The specific calculation steps of carbon emission intensity are listed in Table S4. Finally, the carbon emission intensities of each aircraft type can be found in Tables S5 (China-foreign routes) and S6 (India-foreign routes). This paper does not use code. Any additional information required to reanalyze the data reported in this paper is available from the lead contact upon request.

## Supplemental information

Table S1. Specific steps of data collection, related to experimental procedures

Table S2. The CCD and LTO carbon emissions of China-foreign routes, related to experimental procedures

Table S3. The CCD and LTO carbon emissions of India-foreign routes, related to experimental procedures

Table S4. Specific calculation steps of carbon emission intensity, related to STAR experimental procedures

Table S5. Carbon emission intensities of each aircraft type of China-foreign routes, related to experimental procedures

Table S6. Carbon emission intensities of each aircraft type of India-foreign routes, related to experimental procedures
